# Timing of Treatment for Cytomegalovirus Infection in an Immunocompetent Middle-Aged Woman With Fulminant Ulcerative Colitis: A Case Report

**DOI:** 10.7759/cureus.36092

**Published:** 2023-03-13

**Authors:** Ryuichi Ohta, Tsuyoshi Mishiro, Chiaki Sano

**Affiliations:** 1 Community Care, Unnan City Hospital, Unnan, JPN; 2 Internal Medicine, Unnan City Hospital, Unnan, JPN; 3 Community Medicine Management, Shimane University Faculty of Medicine, Izumo, JPN

**Keywords:** general medicine, anti-neutrophil cytoplasmic antibody, rural hospital, immunocompetent, cytomegalovirus, ulcerative colitis

## Abstract

Viral infections, such as cytomegalovirus (CMV) infection, may affect the clinical course of ulcerative colitis (UC). CMV can cause chronic inflammation of the intestinal mucosa. In inflammatory bowel disease, chronic inflammation caused by CMV can deter the regeneration of the mucosa of the colon. However, the relationship between CMV and inflammatory bowel disease still needs to be clarified, especially in immunocompetent patients, such as younger patients not treated with immunosuppressants. Herein, we describe our experience with a middle-aged immunocompetent female patient diagnosed with fulminant UC positive for myeloperoxidase antineutrophil cytoplasmic antibody (MPO-ANCA). Her initial response to high-dose prednisolone was favorable; however, remission was not achieved. Immunohistochemical staining revealed the presence of CMV. Subsequently, the patient was successfully treated with prednisolone, adalimumab, and azathioprine, along with the anti-CMV treatment comprising valganciclovir. This case shows that the presence of CMV in the mucosa and blood may make patients with UC refractory to immunosuppression; furthermore, the positivity of MPO-ANCA in patients with UC can necessitate the administration of high-dose immunosuppressants to taper the dose of prednisolone.

## Introduction

Ulcerative colitis (UC) is an inflammatory bowel disease that affects the colon and rectum [1]. The clinical course of UC varies depending on the severity of the inflammation. Treatment comprises the administration of disease-modifying medicines, steroids, and immunosuppressants [2]. UC can be controlled by either a single medication or a combination [1]. Aggressive suppression of bowel inflammation is essential for preventing complications, such as perforation, malignancy, and other extraintestinal manifestations [3].

Viral infections, such as those caused by cytomegalovirus (CMV), may influence the clinical course of UC. CMV can cause chronic inflammation of the intestinal mucosa [4]. Chronic inflammation caused by CMV may inhibit the regeneration of the colonic mucosa, especially in patients with inflammatory bowel disease [5]. Some studies have suggested that CMV may play a role in the development or progression of UC [5,6]; however, the relationship between the two still needs to be clarified, especially in immunocompetent patients, such as younger patients not taking immunosuppressants.

The treatment for CMV infection in patients with UC depends on the severity or presence of viremia or an immunocompromised state. Treatment of CMV infection may affect the clinical course of UC depending on the severity of the infection and UC [6]. In cases of severe CMV infections, antiviral medications may suppress viral proliferation and promote UC improvement [6,7]. Here, we describe our experience with a middle-aged, immunocompetent woman diagnosed with fulminant UC. Her initial response to high-dose prednisolone was favorable; however, remission was not achieved. Immunological staining revealed the presence of CMV. Valganciclovir was administered to reduce the prednisolone dose, which led to improvements in the patient’s clinical course. The decision to treat CMV in immunocompetent patients should be based on the presence of viremia, which indicates CMV infection. This case report highlights the importance of balancing the treatment of CMV infection with treatment complications in immunocompetent patients with UC.

## Case presentation

A 53-year-old woman presented to a rural community hospital with chief complaints of loose stools and hematochezia. One month prior to presentation, the patient started having two to three episodes of passing diarrheal stool daily. Two weeks before admission, the frequency of passing loose stools increased to five to six times per week, accompanied by intermittent abdominal pain in the left lower quadrant. She had no symptoms, such as fever, vomiting, or night sweating. The patient visited a primary care physician, where she was diagnosed with bacterial colitis and was prescribed levofloxacin 500 mg daily. However, her abdominal pain and diarrhea symptoms worsened, and a fever of 37.5 °C appeared, so she presented to the hospital. The patient had no history of contact with people with a known infection or anyone with similar symptoms. She did not have any change in eating habits or traveling abroad. The patient had a medical history of hypertension for which she has been prescribed amlodipine 5 mg daily. 

The patient’s vitals at presentation were as follows: blood pressure, 125/80 mmHg; pulse rate, 90 beats/min; body temperature, 38.6 °C; respiratory rate, 20 breaths/min; and oxygen saturation, 96% in room air. The patient was alert and oriented in time, place, and person. Physical examination revealed increased bowel sounds and tenderness in the left lower quadrant of the abdomen, without rebound tenderness. No neurological or musculoskeletal abnormalities were observed. No obvious abnormalities of skin and eyes were observed in the chest. Laboratory test results suggested a hyperinflammatory condition with increased white blood cell count, thrombocytosis, elevated C-reactive protein levels, and a positive result for myeloperoxidase anti-neutrophil cytoplasmic antibody (MPO-ANCA) (Table 1).

**Table 1:** Laboratory data of the patient at presentation eGFR: estimated glomerular filtration rate; CK: creatine kinase; CRP: C-reactive protein; TSH: thyroid-stimulating hormone; Ig: immunoglobulin; HCV: hepatitis C virus; SARS-CoV-2: severe acute respiratory syndrome coronavirus 2; HBs: hepatitis B surface antigen; HBc: hepatitis B core antigen; C3: complement component 3; C4: complement component 4; MPO-ANCA, myeloperoxidase antibody proteinase 3 antibody.

Marker	Level	Reference value
White blood cells	10.3	3.5–9.1 × 10^3^/μL
Neutrophils	84.7	44.0–72.0%
Lymphocytes	8.8	18.0–59.0%
Monocytes	5.9	0.0–12.0%
Eosinophils	0.5	0.0–10.0%
Basophils	0.1	0.0–3.0%
Red blood cells	3.80	3.76–5.50 × 10^6^/μL
Hemoglobin	11.7	11.3–15.2 g/dL
Hematocrit	35.0	33.4–44.9%
Mean corpuscular volume	92.2	79.0–100.0 fl
Platelets	68.1	13.0–36.9 × 10^4^/μL
Erythrocyte sedimentation rate	80	2–10 mm/h
Total protein	6.6	6.5–8.3 g/dL
Albumin	2.2	3.8–5.3 g/dL
Total bilirubin	0.2	0.2–1.2 mg/dL
Aspartate aminotransferase	13	8–38 IU/L
Alanine aminotransferase	11	4–43 IU/L
Alkaline phosphatase	86	106–322 U/L
γ-Glutamyl transpeptidase	44	<48 IU/L
Lactate dehydrogenase	272	121–245 U/L
Blood urea nitrogen	11.0	8–20 mg/dL
Creatinine	0.47	0.40–1.10 mg/dL
eGFR	90	>60.0 mL/min/L
Serum Na	139	135–150 mEq/L
Serum K	3.5	3.5–5.3 mEq/L
Serum Cl	100	98–110 mEq/L
Serum Ca	8.4	3.5–5.3 mg/dL
Serum P	3.6	0.2–1.2 mg/dL
Serum Mg	2.2	1.8–2.3 mg/dL
CK	34	56–244 U/L
CRP	16.18	<0.30 mg/dL
TSH	0.86	0.35–4.94 μIU/mL
Free T4	1.1	0.70–1.48 ng/dL
IgG	1442	870–1700 mg/dL
IgM	41	35–220 mg/dL
IgA	282	110–410 mg/dL
IgE	17	<173 mg/dL
HBs antigen	0.0	IU/mL
HBs antibody	0.00	mIU/mL
HBc antibody	0.00	S/CO
HCV antibody	0.00	S/CO
Syphilis treponema antibody	0.00	S/CO
SARS-CoV-2 antigen	-	
anti-nuclear antibody	40	<40
C3	166	86–164 mg/dL
C4	35	17–45 mg/dL
MPO-ANCA	4.3	<3.5 U/mL
Urine test		
Leukocyte	Negative	Negative
Nitrite	Negative	Negative
Protein	Negative	Negative
Glucose	Negative	Negative
Urobilinogen	Normal	
Bilirubin	Negative	Negative
Ketone	Negative	Negative
Blood	Negative	Negative
pH	6.0	
Specific gravity	1.012	

**Figure 1:**
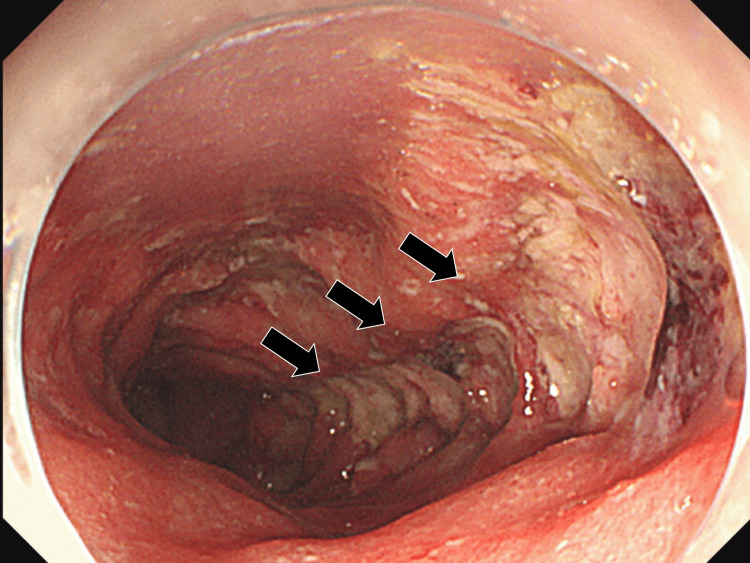
Total colonoscopy revealing erythema, edema, and multiple small ulcers as well as large ulcers (black arrow) with loss of haustral folds

**Figure 2:**
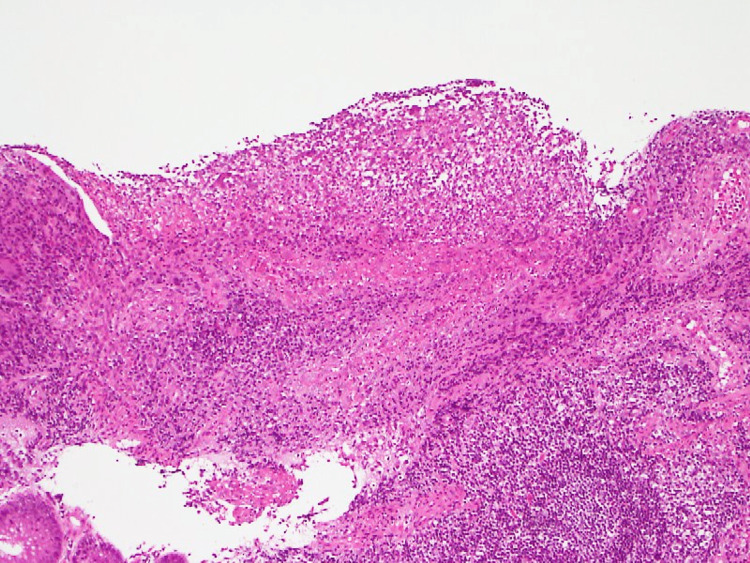
Hematoxylin & Eosin stain of ulcer tissue on the colon showing severe inflammation

Abdominal ultrasonography and computed tomography (CT) showed edema of the wall of the entire colon with mild ascites. We suspected UC and performed a total colonoscopy, which revealed mucus edema, loss of vascularity, spontaneous bleeding, and multiple ulcers from the sigmoid colon to the ileocecal region with severe inflammation (Figure 1). A biopsy of the edge of the ulcer showed multiple infiltrations of neutrophils and lymphocytes without multinucleated giant cells (Figure 2).

 

**Figure 3:**
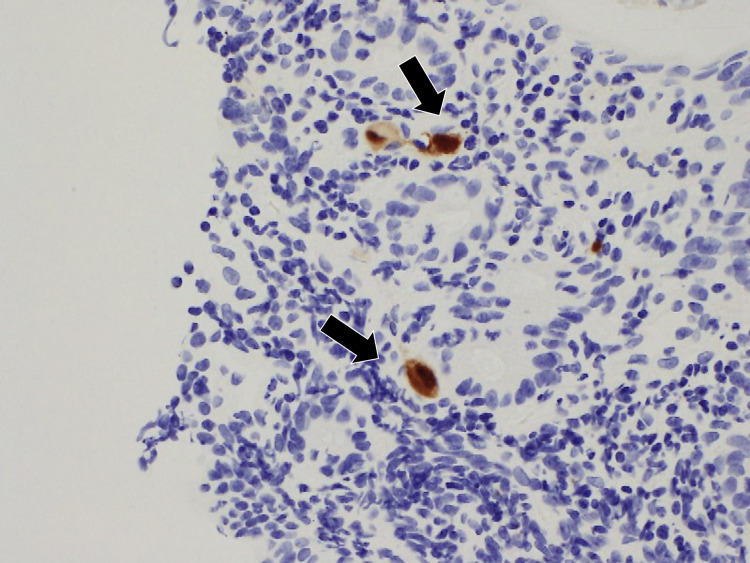
Immunohistochemical stain of the colon revealing cytomegalovirus infection (black arrows)

 Based on these clinical findings, the patient was diagnosed with fulminant UC. She has been treated with oral prednisolone of 60 mg/day and subcutaneous adalimumab of 160 mg followed by 80 mg/2 weeks. Seven days later, the patient’s symptoms were alleviated, but subsequent tapering of prednisolone from 60 to 30 mg in the following two weeks exacerbated the patient’s diarrhea and abdominal pain, and she also developed a mild fever. Therefore, we investigated further by performing additional immunohistochemical staining for CMVs using the colonic tissue obtained during the initial colonoscopy. The results indicated that the mucus cells were infected with CMV (Figure 3).

Although the patient did not have critical liver function abnormalities, we propose that CMV viremia was present and had exacerbated her clinical condition. Positive results for CMV antigenemia confirmed CMV infection. 

We diagnosed the patient with UC complicated by CMV viremia and started oral valganciclovir 1800 mg/day. Diarrhea, abdominal pain, and fever improved over the following days. One week later, a follow-up CMV antigenemia test was negative; therefore, the dose of valganciclovir was tapered to 900 mg/day and was discontinued two weeks later. Prednisolone was also successfully tapered from 30 to 15 mg within one month by adding azathioprine at 75 mg/day. However, the subsequent tapering of prednisolone to 10 mg caused a relapse of fever and diarrhea. Because of the MPO-ANCA positivity, the patient’s condition was considered refractory to treatment; therefore, we planned to taper prednisolone after increasing the dose of azathioprine to 100 mg/day in the outpatient department. She was discharged and resumed her activities of daily living.

## Discussion

Overall, this case shows that the presence of CMV in the mucosa and blood may make patients with UC refractory to immunosuppression; furthermore, UC patients with MPO-ANCA may require high-dose immunosuppressants when tapering prednisolone to a low dose. Rapid tapering of the dose of prednisolone can complicate the clinical course of UC. Tapering prednisolone should be performed promptly when treating UC, as slow tapering can delay the repair of the colonic mucosa [1,8]. In addition, high-dose prednisolone may cause intestinal perforations in patients with severe UC. However, the patient had a UC flare in the present case while the prednisolone dose was tapered. Occult CMV infection and MPO-ANCA are potential causes of such flare; therefore, both conditions should be considered when administering prednisolone to patients with severe UC. 

The optimal treatment for CMV infection in patients with UC remains controversial and depends on the immunological condition of the host. CMV cannot proliferate easily in patients with normal immunity, and infections may be asymptomatic [4]. Because the patient was immunocompetent, we did not initially suspect a CMV infection. However, a refractory state resulting from tapering the dose of prednisolone led to the suspicion of CMV infection, ultimately leading to a diagnosis. CMV may be found in the mucosa of the colon; therefore, when deciding the treatment of CMV infection in patients with severe UC, clarification of the presence of CMV is essential [6,7]. Furthermore, the duration of therapy depends on the disease's severity and the patient’s clinical and virologic response to treatment [9]. In accordance with previous research, we treated the patient until both the symptoms and CMV viremia resolved. The typical duration of therapy is 21 days; however, it may be longer, particularly in patients with tissue-invasive diseases [9,10]. The treatment of CMV infection should be modified based on the viremia and systemic symptoms. The present patient was relatively young and healthy, indicating that antigenemia tests were required for diagnosis and that the dose of valganciclovir could be tapered.

MPO-ANCA positivity may affect the clinical course of patients with UC. The presence of MPO-ANCA indicates the possibility of arterial inflammation, including vasculitis, requiring strong suppression of immunity with immunosuppressants [11]. Our patient was positive for MPO-ANCA; therefore, prednisolone was tapered slowly in conjunction with tumor necrosis factor inhibitors and azathioprine. The quantity of immunosuppressants required varies according to each patient’s response to treatment, and symptoms change when the prednisolone dose is tapered [12]. In our case, azathioprine 100 mg/day and adalimumab 80 mg/2 weeks were administered, allowing the tapering of prednisolone to 15 mg/day. However, further tapering resulted in a relapse of symptoms. This experience suggests that in cases of UC with positive MPO-ANCA results, clinicians should note the patients’ subtle symptoms and taper prednisolone slowly while waiting to observe the effect of azathioprine, which can affect human immunity over a long period. Furthermore, it should be noted that vague symptoms, such as mild fever, can initiate a flare of various autoimmune diseases [13].

## Conclusions

Our case shows that the presence of CMV in the mucosa and blood may render patients with UC refractory to immunosuppression. CMV in the blood of refractory patients should be promptly treated with antiviral drugs. Furthermore, MPO-ANCA-positive patients with UC may require high-dose immunosuppressants to lower the dose of prednisolone. Thus, rheumatologists should treat patients with UC in accordance with the findings of their autoimmune panels, such as ANCA, from the initiation of treatment to anticipate a refractory clinical course.
